# Honorary authorship in health sciences: a protocol for a systematic review of survey research

**DOI:** 10.1186/s13643-022-01928-1

**Published:** 2022-04-04

**Authors:** Reint Meursinge Reynders, Gerben ter Riet, Nicola Di Girolamo, Mario Malički

**Affiliations:** 1 Department of Oral and Maxillofacial Surgery, Amsterdam University Medical Center (Amsterdam UMC) Location AMC, Meibergdreef 9, 1105 AZ Amsterdam, The Netherlands; 2 Studio di Ortodonzia, Via Matteo Bandello 15, 20123 Milan, Italy; 3 Urban Vitality Centre of Expertise, Amsterdam University of Applied Sciences, Amsterdam, The Netherlands; 4 Department of Cardiology, Amsterdam University Medical Center (Amsterdam UMC) Location AMC, Meibergdreef 9, 1105 AZ Amsterdam, The Netherlands; 5 Center for Veterinary Health Sciences, Oklahoma State University, 2065 W, Farm Road, Stillwater, Oklahoma 74078 USA; 6 EBMVet, Via Sigismondo Trecchi 20, 26100 Cremona, CR Italy; 7 Meta-research Innovation Center a Stanford (METRICS), Stanford University, Stanford, USA

**Keywords:** Honorary authorship, Guest authorship, Gift authorship, Contribution disclosure, ICMJE, Publication ethics, Scientific misconduct, Research integrity, Research transparency, Surveys

## Abstract

**Background:**

Honorary authorship refers to the practice of naming an individual who has made little or no contribution to a publication as an author. Honorary authorship inflates the output estimates of honorary authors and deflates the value of the work by authors who truly merit authorship. This manuscript presents the protocol for a systematic review that will assess the prevalence of five honorary authorship issues in health sciences.

**Methods:**

Surveys of authors of scientific publications in health sciences that assess prevalence estimates will be eligible. No selection criteria will be set for the time point for measuring outcomes, the setting, the language of the publication, and the publication status. Eligible manuscripts are searched from inception onwards in PubMed, Lens.org, and Dimensions.ai. Two calibrated authors will independently search, determine eligibility of manuscripts, and conduct data extraction. The quality of each review outcome for each eligible manuscript will be assessed with a 14-item checklist developed and piloted for this review. Data will be qualitatively synthesized and quantitative syntheses will be performed where feasible. Criteria for precluding quantitative syntheses were defined a priori. The pooled random effects double arcsine transformed summary event rates of five outcomes on honorary authorship issues with the pertinent 95% confidence intervals will be calculated if these criteria are met. Summary estimates will be displayed after back-transformation. Stata software (Stata Corporation, College Station, TX, USA) version 16 will be used for all statistical analyses. Statistical heterogeneity will be assessed using Tau^2^ and Chi^2^ tests and *I*^2^ to quantify inconsistency.

**Discussion:**

The outcomes of the planned systematic review will give insights in the magnitude of honorary authorship in health sciences and could direct new research studies to develop and implement strategies to address this problem. However, the validity of the outcomes could be influenced by low response rates, inadequate research design, weighting issues, and recall bias in the eligible surveys.

**Systematic review registration:**

This protocol was registered a priori in the Open Science Framework (OSF) link: https://osf.io/5nvar/.

**Supplementary Information:**

The online version contains supplementary material available at 10.1186/s13643-022-01928-1.

## Background

Authorship has been called ‘the currency of an academic career’ [1]. As with all currencies, they can be obtained through hard work, but also through less transparent means which has resulted in widely reported honorary authorship disputes [2–4]. Honorary authorship (HA) violates ethical publication principles and skews the output estimates of both honorary authors and those that merit authorship. This manuscript presents a protocol for a systematic review to assess the prevalence of 5 HA issues in the health sciences.

HA refers to authorship assigned to individuals that should not have been included as authors of a publication, because they made no or insufficient contributions to qualify as authors (Table 1). Discussions on assigning authorship can be complicated especially if the power between stakeholders is imbalanced, e.g., junior versus senior scientists, mentees versus mentors, PhD students versus promotors, individuals with administrative power versus those without [8, 9]. Senior scientists assign specific tasks to junior scientists to complete their research projects, and indirectly, to extend their number of publications. This output is important for senior scientists, because the number of published papers, particularly in journals with high impact factors, is considered a key element of their CV and often a predominant measure in the decisional process for hiring, promotion, and tenure of researchers [10–12]. Power games between senior and junior scientists can lead to authorship disputes and to not-merited assignments of authorship, i.e., HA. In our personal experience as research integrity teachers, the irritation among junior researchers about these practices is a recurrent theme. A salient example of HA is automatically assigning authorship to a senior member, often the head of a department [13, 14]. Explicit academic bullying by scientists of junior team members on authorship issues is also a serious concern [15, 16]. Kovacs [9] reports on the phenomenon of publication cartels, i.e., groups of researchers that frequently publish articles together with the explicit agreement that the HAs will be granted reciprocally.

**Table 1:** Glossary of terms

Term	Definition
Survey [5]	Wikipedia [5] defines a survey as follows: ‘In research of human subjects , a survey is a list of questions aimed at extracting specific data from a particular group of people’.
Health sciences [6]	Wikipedia [6] defines ‘health sciences’ as ‘are those sciences which focus on health , or health care , as core parts of their subject matter. These two subject matters relate to multiple academic disciplines, (and as such) both STEM disciplines, as well as emerging patient safety disciplines (such as social care research ), and are both relevant to current health science knowledge.’
Honorary authorship	Refers to authorship assigned to individuals that should not have been included as authors of a publication, because they made no or insufficient contributions to qualify as authors.
ICMJE-defined criteria for authorship [7]	The ICMJE recommends that authorship is based on the following 4 criteria: 1) ‘Substantial contributions to the conception or design of the work; or the acquisition, analysis, or interpretation of data for the work; AND 2) Drafting the work or revising it critically for important intellectual content; AND 3) Final approval of the version to be published; AND 4) Agreement to be accountable for all aspects of the work in ensuring that questions related to the accuracy or integrity of any part of the work are appropriately investigated and resolved.’
ICMJE-based honorary authorship	The perception/opinion of the surveyee that that one or more of the co-authors did not meet the criteria for authorship of the ICMJE.
Perceived honorary authorship	The perception/opinion of the surveyee that one or more of the co-authors should not have been included as author(s) of a publication, because they made no or insufficient contributions to qualify as authors.
**Review item 1:** Researchers perceiving other co-author(s) as honorary author(s) on a publication	The perception/opinion of the surveyee that one or more of the co-authors should not have been included as author(s) of a publication, because they made no or insufficient contributions to qualify as authors.
**Review item 2:** Researchers having been approached by others to include honorary author(s) on a publication	The surveyee has been approached by others to include honorary author(s) on a publication.
**Review item 3:** Researchers admitting being an honorary author on a publication	Admitting by the surveyee that he/she should not have been included as an author of a publication, because he/she made no or insufficient contributions to qualify as an author.
**Review item 4:** Researchers admitting adding an honorary author(s) on a publication	Admitting by the surveyee that he/she has included honorary author(s) on a publication.
**Review item 5:** Researchers admitting having approached others to include honorary author(s) on a publication	Admitting by the surveyee that he/she has approached others to include honorary authors on a publication.

The prevalence of HA is commonly [17–19] measured as perceived HA or International Committee of Medical Journal Editors (ICMJE)-based HA [7]. These and other key terms are defined in Table 1. The ICMJE has developed their authorship criteria to reduce authorship disputes and to discourage scientists from granting credit to researchers who do not merit authorship.

However, the prevalence of ICMJE-based HA could be misleading for the following reasons: (1) not all surveyees might be aware of the ICMJE criteria or their definitions when completing the surveys (2) the ICMJE criteria have changed over the years ([20] (3) the risk of subjective interpretation by surveyees of the various terms in the criteria such as ‘substantial’, ‘critically’, ‘important’, ‘appropriately’ (Table 1). In this planned systematic review, we will assess HA issues for both perceived as well as ICMJE-based HA and how they change over time. These issues are summarized under review items 1–5 in Table 1. The latter 3 review items report on self-admitted HA issues by the surveyee. These statistics are important, because surveyees are likely to underreport their own misconduct and overestimate those of co-authors. This was found in meta-analyses of surveys on research integrity issues such as scientists admitting plagiarism [21] or having fabricated or falsified data [22].

A scoping search in PubMed was undertaken by 2 reviewers (RMR and NDG) to assess whether previous reviews on our research questions have been published. Only 2 earlier reviews were identified, but these manuscripts gave narrative reviews on authorship issues [23], publication practices and responsible authorship [24] or systematically reviewed other authorship issues [25]. On the basis of the findings in our scoping search, we concluded that HA merits a systematic review and that no prior systematic reviews seem to have covered our research questions. Understanding the magnitude of the various HA issues under review may accelerate the design of future studies and bring ideas on how to counteract HA. These strategies could reduce stress among a broad spectrum of researchers, reduce time wasted on authorship issues, boost meritocracy, reduce power games, implement the fining of those guilty of HA, improve collaboration between researchers, and possibly increase the overall happiness in research and academic settings. This is important, because a recent survey of almost 4000 scientists showed that the majority (55%) had a negative impression of the research culture they are working in [26].

## Objectives

For the primary objectives of this systematic review the prevalence of the following 2 review items will be assessed (Table 1):

### Review item 1

Researchers perceiving other co-author(s) as honorary author(s) on a publication.

### Review item 2

Researchers having been approached by others to include honorary author(s) on a publication.

The secondary research objectives will focus on self-declared HA issues. For these objectives the prevalence of the following review items will be assessed (Table 1).

### Review item 3

Researchers admitting being an honorary author on a publication.

### Review item 4

Researchers admitting adding an honorary author(s) on a publication.

### Review item 5

Researchers admitting having approached others to include honorary author(s) on a publication.

All five review items will be assessed for perceived-and ICMJE-based HA separately. We will also assess how outcomes change over time.

## Methods

We used The Preferred Reporting Items for Systematic review and Meta-Analysis Protocols (PRISMA-P) 2015 statement [27, 28] and the Joanna Briggs Reviewers manual for systematic reviews of prevalence and incidence [29, 30] to develop and report this protocol. This manuscript is registered in the Open Science Framework (OSF) [31] and not in PROSPERO [32], because our research questions do not meet the inclusion criteria for the latter register.

### Eligibility criteria

Table 2 presents eligibility criteria for domain, study designs, participants, survey instruments, outcomes, time point, setting, language, publication status, and publication dates [27, 28].

**Table 2:** Inclusion and exclusion criteria

Item	Inclusion criteria	Exclusion criteria
**Domain**	• Health sciences as defined in Table 1.	
**Study designs**	• Studies including at least one survey according to its definition in Table 1.	• Surveys in which it was unclear what questions were used to assess review items 1–5 (Table 1), i.e., surveys which did not report or whose authors were unreachable or did not provide exact questions asked in the survey
**Participants**	• Any author on the author list of a scientific publication, e.g., first, last, corresponding author, that was invited to participate in a survey on at least one of our authorship items.	
**Survey instruments**	• Surveys based on questionnaires for self-completion. • Surveys administered by email, internet platforms, by post, or by hand. • We will only consider closed surveys, i.e., surveys open to a specific sample of participants selected by the investigators. • Surveys with or without incentives to complete it.	• Focus groups discussions and one-to one interviews.
**Outcomes**	• One or more of the outcomes on authorship issues listed in our objectives for review items 1–5 (Table 1). • Both self-and non-self-reported outcomes on authorship issues.	• Outcomes that were not reported as prevalence statistics or were not given in a format that such statistics could be calculated.
**Time point**	• Any time point for measuring outcomes will be eligible, i.e., we will not set exclusion criteria whether an article on which the surveyee was questioned was published 1, 2, 3 etc. years previously.	
**Setting**	• Any	
**Language**	• Any	
**Publication status**	• Peer-and non-peer-reviewed manuscripts.	
**Publication dates**	• Articles published from bibliography inception onwards.	

### Information sources and search strategy

PRISMA-S was consulted to report the literature search [33], as well as a previous systematic review on the meaning, ethics and the practices of authorship published by Marušić et al. [25], which used “authorship” as the only word for their search strategy. We will perform a systematic search in the following electronic databases from inception onwards: PubMed, Lens.org, and Dimensions.ai, with no language or date filters, but applying health sciences filters for Lens.org and Dimensions.ai for full search strategies. Additionally, all included papers will be checked for additional refences mentioned in their introduction or discussion sections. The full search strategies are presented in Additional file 1. These strategies were developed by one of the authors (MM) with the aim to capture all surveys on authorship, as honorary authorship might have been only one question in those surveys, or a secondary outcome that was not mentioned in the abstract. Additionally, these searches captured all studies we identified in the pilot, expect 4 that did not have indexed abstracts and were short reports. These 4 however were referenced in studies we identified, so would have been captured by checking the introduction or discussions or papers, which, as mentioned above, we will do for all included studies.

### Data management and selection process

Two authors (RMR and NDG) will be calibrated a priori through pilot tests. These investigators will screen titles and abstracts independently to select potentially relevant papers. Identified records will be imported in Rayyan [34] and duplicate records will be removed. Rayyan will be subsequently used for the initial screening of titles and abstracts to identify potentially relevant papers. In the initial search phase, the selection of studies will be overinclusive [35]. Full texts of potentially eligible manuscripts will be retrieved and assessed for eligibility. We will implement Cochrane’s strategies to identify multiple reports from the same study [35]. We will further assess whether relevant studies were retracted, fraudulent, or whether errata or comments were given [35]. Authors will be contacted to verify the eligibility of their surveys.

After these assessments we will make final decisions on study eligibility. Reference lists and citing articles of the selected eligible papers will also be crosschecked for additional relevant studies. A list of excluded studies with rationales for exclusion will be given. This list will focus on all studies that initially appear eligible, but after further inspection do not meet the eligibility criteria [35]. Our search strategy will not include a filter for manuscripts published in the health sciences. All non-health science manuscripts that assess authorship issues will also be presented in the list of excluded studies with the reason for exclusion. The study selection procedures will be reported in a PRISMA flow chart [36, 37].

### Data collection process and data items

For the development of our data extraction forms we consulted the reporting checklists of the Strengthening the Reporting of Observational Studies in Epidemiology (STROBE) statement for reporting cross-sectional studies [38], the Checklist for Reporting Results of Internet E-Surveys (CHERRIES) for reporting the survey [39, 40], and the Checklist for polls by Bethlehem [41]. To ensure consistency in the data collection process, we will conduct calibration exercises for both data extractors a priori. These investigators (RMR and NDG) will independently extract the pertinent data from each eligible study. The pilot-tested data collection forms are listed in Additional file 2A. A description of each data item is given in these forms. Disagreement between investigators on study inclusion and issues regarding data extraction will be resolved through discussions. Persistent disagreements will be resolved through arbitration by a third author (GTR) or through contacting the authors of the pertinent publications [28]. We will document when and why this was deemed necessary.

### Outcomes and prioritization

The prevalence of researchers perceiving other co-author(s) as honorary author(s) on a publication and the prevalence of researchers having been approached by others to include honorary author(s) on a publication will be the primary outcomes. The prevalence of researchers admitting being an honorary author on a publication, the prevalence of researchers admitting adding an honorary author(s) on a publication, and the prevalence of researchers admitting having approached others to include honorary author(s) on a publication will be the secondary outcomes. The definitions of these outcomes and the pertinent numerators and denominators are given in Table 3. The various response rates measured are also reported in these tables. These outcomes will be calculated separately for perceived HA and ICMJE-based HA (Table 1). We included ICMJE-based HA in this updated version of our protocol, because our initial screening and data extraction revealed that many surveys operationalize ICMJE-based HA. We are convinced that including ICMJE-based HA outcomes will considerably strengthen our systematic review. For each included survey we will report the exact question on which each outcome was based.

**Table 3:** Definition of response rates and primary and secondary outcomes

Outcome	Definition
Number of emails with questionnaires on HA issues sent (N1)	The total number of emails with questionnaires on HA issues sent.
Number of emails with questionnaires on HA issues not bounced (N2)	The total number of emails with questionnaires on HA issues sent that had surveyees with valid email addresses.
Number of questionnaires for which the surveyee was available (N3)	The total number of emails with questionnaires sent to assess HA issues with surveyees with valid email addresses and for which the surveyee was available. Unavailability can be the result of, e.g., automated responses such as ‘out of office’, ‘study leave’, ‘on strike’, ‘vacation leave’, ‘maternity leave’
Number of partly or completely answered questionnaires (N4)	The total number of questionnaires on HA issues received back in which the questions were answered (either partial or completely).
Number of completely answered questionnaires (N5)	The total number of questionnaires on HA issues received back in which all questions were answered.
Overall response rates in questionnaires on HA issues	N4 or N5/N1, N2, or N3
Number of questionnaires that answered the question on review item 1^a^ (N6)	The total number of questionnaires received back in which the question on review item 1^a^ was answered.
Response rate on review item 1^a^	N6/N1, N2, N3, N4 or N5
Number of questionnaires in which the respondents reported review item 1^a^ (N7)	The number of questionnaires in which the respondents reported review item 1^a^, i.e., perceiving other co-author(s) as honorary author(s) on a publication.
Prevalence of review item 1^a^ **(primary outcome)**	N7/N6
Number of questionnaires that answered the question on review item 2^b^ (N8)	The total number of questionnaires received back in which the question on review item 2^b^ was answered.
Response rate on review item 2^b^	N8/N1, N2, N3, N4, or N5.
Number of questionnaires in which the respondents reported review item 2^b^ (N9)	The number of questionnaires in which the respondents reported review item 2^b^, i.e., having been approached by others to include honorary author(s) on a publication.
Prevalence of review item 2^b^ **(primary outcome)**	N9/N8
Number of questionnaires that answered the question on review item 3^c^ (N10)	The total number of questionnaires received back in which the question on review item 3^c^ was answered.
Response rate on review item 3^c^	N10/N1, N2, N3, N4, or N5
Number of questionnaires in which the respondents reported review item 3^c^ (11)	The number of questionnaires in which the respondents reported review item 3^c^, i.e., admitting being an honorary author on a publication.
Prevalence of review item 3^c^ **(secondary outcome)**	N11/N10
Number of questionnaires that answered the question on review item 4^d^ (N12)	The total number of questionnaires received back in which the question on review item 4^d^ was answered.
Response rate on review item 4^d^	N12/N1, N2, N3, N4, or N5.
Number of questionnaires in which the respondents reported review item 4^d^ (N13)	The number of questionnaires in which the respondents reported review item 4^d^, i.e., admitting adding an honorary author(s) on a publication.
Prevalence of review item 4^d^ **(secondary outcome)**	N13/N12
Number of questionnaires that answered the question on review item 5^e^ (N14)	The total number of questionnaires received back in which the question on review item 5^e^ was answered.
Response rate on review item 5^e^	N14/N1, N2, N3, N4 or N5
Number of questionnaires in which the respondents reported review item 5^e^ (15)	The number of questionnaires in which the respondents reported review item 5^e^, i.e., admitting having approached others to include honorary author(s) on a publication.
Prevalence of review item 5^e^ **(secondary outcome)**	N15/N14

^a^Review item 1: Researchers perceiving other co-author(s) as honorary author(s) on a publication

^b^Review item 2: Researchers having been approached by others to include honorary author(s) on a publication

^c^Review item 3: Researchers admitting being an honorary author on a publication

^d^Review item 4: Researchers admitting adding an honorary author(s) on a publication

^e^Review item 5: Researchers admitting having approached others to include honorary author(s) on a publication

### Assessment of methodological quality

The methodological quality of each survey will be assessed with a survey checklist of 14 items that was tailored to our research questions on HA issues. To develop this quality checklist, we conducted pilot tests and consulted existing appraisal tools [29, 30, 39–46]. To rate the overall confidence in the results of the survey, we adopted the rating scheme reported for the AMSTAR 2 critical appraisal tool [47], which is based on an assessment of critical-and non-critical items. As in the AMSTAR 2 tool we labeled 7 of the 14 items of our quality checklist as ‘critical’, because we believe that these items can critically affect the validity of the outcomes of a survey. In congruence with AMSTAR 2 we will assign 4 ratings: ‘high’, ‘moderate’, ‘low’, and ‘critically low’ rating of the overall confidence in the results of the survey. These ratings reflect how non-implementation of one or more of these quality safeguards possibly have impacted bias of the results of the survey. A detailed description of this survey checklist and guidance on the rating scheme are given in Additional file 2B. We will list the critical appraisal scores for each included survey and will calculate the prevalence of yes scores (all yes scores/number of surveys) for each critical appraisal question (Additional file 2C) [48]. Two reviewers (RMR and NDG) will independently implement the methods reported for the 14-item quality checklist. This tool will be used for each of the 5 outcomes of this review. A third reviewer (GTR) will be consulted in the case of persistent disagreements. We will report when and why these consultations were necessary. Four surveys will be used a priori to calibrate the operators.

### Data synthesis

#### Criteria for a quantitative synthesis

Outcomes in this review are prevalence statistics (proportions), which can be quantitatively synthesized. We may preclude meta-analyses for the following scenarios: (1) only 1 or no included studies, (2) very different definitions of outcomes, (3) incomplete reporting of proportions, (4) biased evidence such as ‘low’, and ‘critically low’ ratings of the overall confidence in the results of the survey, (5) explained and unexplained heterogeneity [49]. We will consider a *I*^2^ larger than 50% as an approximate rule of thumb for not conducting meta-analyses. When applying this rule we will consider that the value of *I*^2^ depends on the direction and magnitude of the outcomes and the strengths of the evidence for the identified heterogeneity [50]. Prior to precluding meta-analyses we will assess if solutions are possible for dealing with one or more of these limiting criteria [49].

#### Summary measures for a quantitative synthesis

The prevalence proportions and their exact (method = Wilson) 95% confidence limits across studies will be visually displayed in a forest plot. If the criteria for calculating a pooled estimate are met, we will pool the proportions and report them with their 95% confidence intervals. Double arcsine transformation will be performed prior to any statistical pooling. Summary estimates will be displayed after back-transformation [51]. These calculations will be performed using the metaprop command in Stata 16 [52]. We plan to implement a random-effects model, because between-study variance is expected.

#### Unit of analysis issues

To address unit-of-analysis issues, we will assess in each study whether surveyees underwent more than one survey, e.g., surveys conducted at multiple time points on the same individuals.

#### Dealing with missing data

To address missing data, we will contact the corresponding author and the author that was acknowledged as involved in the statistical analysis of the pertinent research studies. Such authors will be contacted by email. A first reminder will be sent one week after the first one and a second after 2 weeks. We will then wait for 2 weeks and accept the data to be missing and proceed.

#### Assessment of heterogeneity

We will assess the presence and the extent of heterogeneity. In the forest plots we will assess the overlap of the confidence intervals for the results of the individual surveys. We will calculate Tau^2^ (estimate of between study variance) and Chi^2^ tests to measure statistical heterogeneity and *I*^2^ to quantify inconsistency [50].

#### Investigation of heterogeneity

We will assess survey-related and methodological diversity [50]. We will conduct subgroup analyses and meta-regression to investigate heterogeneity. The following explanatory variables for these investigations will be considered for these analyses:

Survey-related diversity

- The type of authors that were surveyed, i.e., first authors versus corresponding or any other author in between (Table 1)
- Career levels of the surveyee, e.g., PhD students, post doctorates, department chairpersons
- The field of research on which the surveyee was interviewed, e.g., radiology, urology, dentistry
- The country of the first institution listed in the manuscript
- The journal impact factor, e.g., above or below 5.00
- The year of conducting the survey
- The method of survey delivery, e.g., administered by email, internet platforms, by post
- Anonymity of the surveyee
- Definitions of HA
- The magnitude of the response rates, e.g., above or below 25%

Methodological diversity

- The method of sampling, e.g. randomly selected or not
- Sample size
- The time point for measuring outcome (the recall period), e.g., before or after 1 year of publication of the manuscript on which authors were surveyed.
- Study quality, e.g., low, moderate, or high quality
- Response rate

We will visually display the individual effects for each planned prevalence outcome in stratified forest plots according to these explanatory variables. To avoid misinterpretation of the findings, we will not give the combined effect estimate in these plots if the criteria for a meta-analysis are not met [53].

We will also build generalized linear mixed models (GLMMs) to assess what factors contribute to each of the five review outcomes on HA issues. For each study that reported prevalence data on these issues, the following data will be extracted and tabulated in an electronic spreadsheet: number of respondents and the number of individuals reporting each outcome. Each individual respondent was listed as a row in the electronic spreadsheet. For the GLMMs, the presence/absence of an HA issue will be the dependent variable and all the explanatory variables reported previously as the predictors. Study ID will be included as a random effect, in order to account for clustering at a study level. The other variables will be included as fixed effect. The linearity assumption of the continuous variables will be assessed as described elsewhere [54–56]: (1) the variables will be binned in quartiles; (2) the multivariate model will be fitted replacing the continuous variables with the four-level binned variables; (3) log odds of the upper three quartiles (the lower quartile will be used as indicator) will be plotted versus the respective quartile midpoints; (4) the four plotted points will be connected with straight lines and the plot will be visually inspected for linearity. Specific categories of categorical predictors will depend on categories used by primary investigators in their surveys. Depending on the total number of individual response data available, different modeling approach will be employed to include/exclude independent variables and avoid overfitting of the model. We will apply the same methods for additional explanatory variables that will be identified during the review process. We will explain in the final review why these variables were added.

#### Qualitative synthesis

We will conduct a systematic narrative synthesis whether quantitative syntheses will be possible or not.

Tables will be developed to report the characteristics of included surveys as reported in Additional file 2A. We will consult these tables for the data synthesis and assess relationships and diversity within and between surveys.

### Sensitivity analysis

We will undertake sensitivity analyses to investigate the impact of certain decisions on the outcomes of the systematic review. Such decisions could refer to the searching for surveys, the choice of certain eligibility criteria such as the characteristics of the survey design, methods to be used for the analysis (fixed-effect or random-effects methods), and the quality of the included surveys. However, which specific issues to explore in sensitivity analyses will be decided during the review process [50]. We will report the findings of these analyses in a summary table [50].

### Meta-biases

We will assess the presence of non-reporting biases. Such biases occur when results are missing from surveys that should have been included in the syntheses of the review [57, 58]. Non-reporting biases can come in many forms such as publication, time-lag, language, citation, multiple publication, location, and selective (non-) reporting bias [57]. We will implement a variety of strategies to address the risk of non-reporting biases such as:

1. Use a broad spectrum search strategy and searching studies in a wide body of search engines.
2. Assess the availability of study protocols and registers and if available assess whether the planned outcomes in the protocols are the same as those reported in the included studies.
3. Contacting authors regarding issues such as multiple publications of research data, information on the availability of protocols, unpublished, or ongoing studies.
4. After the implementation of these 3 strategies we will adopt the 6-step Cochrane framework for assessing risk of bias as a result of missing results in a synthesis, e.g., a meta-analysis [58]. For the sixth step of this protocol, an overall judgment on the risk of bias as a result of missing results for each synthesis will be given.

### Confidence in the cumulative evidence

We will present summary of findings tables that report the magnitude of the outcome, the certainty, or quality of evidence for each primary and secondary outcome, and other key data (Additional file 2B and C). We will adopt the GRADE approach to assess the overall certainty of the body of evidence for each outcome that was sought in this review [59]. For this approach we will assess: (1) bias in the included surveys, i.e., the overall confidence in the results of each survey based on our 14-item quality checklist (Additional file 2B), (2) heterogeneity or inconsistency of results, (3) indirectness of evidence, (4) imprecision of results, and (5) publication bias [59]. The GRADE approach assigns four levels of certainty: ‘high’, ‘moderate’, ‘low’, and ‘very low certainty’ [59]. The rationale for assigning these ratings will be given. Guidance for grading the certainty or quality of evidence is reported in Additional file 2B.

### Differences between the protocol and the review

Any differences between what is described in this protocol and the methods implemented in the final systematic review will be reported with rationale. We will also explain, if possible, the potential consequences of these changes for the direction, magnitude, and the validity of the results [60].

## Discussion

### What will this research study investigate?

We will search the literature for surveys in the health sciences that assessed the prevalence of a variety of HA issues. In addition, we will compare the statistics for self-and non-self-reporting by the respondents of the surveys. Our scoping searches showed that no previous systematic reviews have assessed our research questions.

### What are its strengths?

The strengths of this review include (1) the broad-spectrum search strategy and information sources, (2) contacting authors to verify the eligibility of surveys and to retrieve additional research data on these surveys such as obtaining questionnaires, (3) pre-registration of the protocol in Open Science Framework, (4) the use of a 14-item quality checklist developed and piloted for the review questions, (5) a research team that covers all disciplines for conducting systematic reviews including topic expertise, and (6) assessing both self-and-non-self-reported authorship issues.

### What are its limitations?

However, this review could also have some limitations. We expect that the validity of its findings could be conditioned by factors such as the magnitude of the response rates, sample size, differences in characteristics of the responders and non-responders, poor reporting, non-anonymous surveys, survey-related and methodological diversity, missing data, quality of the data analysis, and the intrinsic nature of surveys, e.g., the risk of recall bias, in particular when much time has passed between the completion of a research study and the survey on the pertinent study.

### Why this research study is important

HA causes inflated output estimates of honorary authors and deflates the importance of the work done by authors who truly merit authorship. These distorted numbers could subsequently have effects on (1) the careers and riches of these stakeholders, (2) the order of authors in the author list, (3) peer review (big names in the author list), (4) norms perception by others, e.g., juniors, in science.

The outcomes of this research study will provide insight in the magnitude of the prevalence of HA issues.

High prevalence statistics could direct new research studies on these issues and accelerate the development and implementation of tailored strategies to address them such as education in publication ethics [61, 62], redefining and fine-tuning criteria for authorship and adopting the Contributor Roles Taxonomy (CRediT) [63], and possibly even devalue authorship. However, high prevalence statistics could also be used as a confirmation by violators of authorship issues that HA is common, so why should I behave differently? Deans of universities, research directors, editors, peer reviewers, authors, publishers, and research sponsors are just some of the key players that can make a difference. If they are willing to address HA issues they could reduce stress among researchers, reduce time wasted on authorship issues, boost meritocracy, improve collaboration between researchers, and possibly even the overall quality of life at universities and other research institutes. Addressing HA issues could also be an important step in improving the trust between the general public and the research community [64].

## Supplementary Information

**Additional file 1.** Search strategy.

**Additional file 2.** Data collection forms, quality checklists, and summary of findings tables.

## Data Availability

All data generated or analyzed for this research study will be reported in the systematic review.

## Abbreviations

CHERRIES: Checklist for Reporting Results of Internet E-Surveys
CRediT: Contributor Roles Taxonomy
GLMM: Generalized linear mixed model
HA: Honorary Authorship
ICMJE: International Committee of Medical Journal Editors
ORCID: Open Researcher and Contributor ID
OSF: Open Science Framework
STROBE: Strengthening the Reporting of Observational Studies in Epidemiology

## Declarations

Ethics approval and consent to participate
