# Supplementary material for: Honorary authorship in health sciences: a protocol for a systematic review of survey research
Source: Syst Rev. 2022 Apr 4;11:57. doi: 10.1186/s13643-022-01928-1 (PMC8978359; doi:10.1186/s13643-022-01928-1)
Supplement: Supplementary file 1 — Additional file 1. Search strategy. [file 13643_2022_1928_MOESM1_ESM.docx]

**Additional file 1**

**Search strategies for PubMed, Lens.org, and Dimensions.ai**

**PubMed**

| **Date search:** April 12 2021  **Search strategy:** (contributorship[Title/Abstract] OR authorship[Title/Abstract]) AND (survey[Title/Abstract] OR questionnaire[Title/Abstract])  **Link:**<https://pubmed.ncbi.nlm.nih.gov/?term=%28contributorship%5BTitle%2FAbstract%5D+OR+authorship%5BTitle%2FAbstract%5D%29+AND+%28survey%5BTitle%2FAbstract%5D+OR+questionnaire%5BTitle%2FAbstract%5D%29>  **Results:** 291 |
| --- |

**Lens.org**

| **Date search:** April 12 2021  **Search strategy:** (title:(authorship OR contributorship) OR abstract:(authorship OR contributorship)) AND (title:(survey OR questionnaire) OR abstract:(survey OR questionnaire))  Filters: Field of Study = (Medicine, Medical education, Family medicine, Alternative medicine, Nursing, Nurse education )  **Link:** <https://www.lens.org/lens/search/scholar/list?q=(title:(authorship%20OR%20contributorship)%20OR%20abstract:(authorship%20OR%20contributorship))%20AND%20(title:(survey%20OR%20questionnaire)%20OR%20abstract:(survey%20OR%20questionnaire))&p=0&n=10&s=_score&d=%2B&f=false&e=false&l=en&authorField=author&dateFilterField=publishedYear&orderBy=%2B_score&presentation=false&stemmed=true&useAuthorId=false&fieldOfStudy.must=Medicine&fieldOfStudy.must=Medical%20education&fieldOfStudy.must=Family%20medicine&fieldOfStudy.must=Alternative%20medicine&fieldOfStudy.must=Nursing&fieldOfStudy.must=Nurse%20education>  **Results:** 391 |
| --- |

**Dimensions.ai**

| **Date search:** April 12 2021  **Search strategy:** (contributorship or authorship) and (survey or questionnaire)  Filter: Fields of Research: Medical and Health Sciences  **Link:**  <https://app.dimensions.ai/discover/publication?search_mode=content&search_text=(contributorship%20or%20authorship)%20and%20(survey%20or%20questionnaire)&search_type=kws&search_field=full_search&and_facet_for=2211>  **Results:** 563 |
| --- |
