# Supplementary material for: Honorary authorship in health sciences: a protocol for a systematic review of survey research
Source: Syst Rev. 2022 Apr 4;11:57. doi: 10.1186/s13643-022-01928-1 (PMC8978359; doi:10.1186/s13643-022-01928-1)
Supplement: Supplementary file 2 — Additional file 2. Data collection forms, quality checklists, and summary of findings tables. [file 13643_2022_1928_MOESM2_ESM.docx]

**Table of contents for additional file 2**

| **Page(s)** | **Additional file item** | **Description** |
| --- | --- | --- |
| 1-5 | Additional file 2A | Data collection forms |
| 6-11 | Additional file 2B | Quality checklist for surveys on Honorary Authorship (HA) items,  Guidance for completing the quality checklist for surveys on HA issues, Guidance for rating the overall confidence in the results of a survey |
| 12 | Additional file 2C | Summary of findings tables |
| 13 | References | References for additional file 2 |

**Additional file 2A**

**Data collection forms**

All entries of the data collection forms will be collected in Excel spreadsheets.

**Table 1. Data collection forms at the study level in surveys on HA issues***

| **Entry** | **Description** |
| --- | --- |
| Year | Report the year of the publication of the article. |
| Reference | Report the full reference. |
| Journal | Report the name of the pertinent journal. |
| IF(year) | Report the impact factor of the journal. We will report the latest impact factor and the year in which this impact factor was assigned. |
| Language | Report the language of the article. |
| Number of authors | Report the number of authors in the article. |
| Affiliation | Report the type of the first affiliation, i.e., university, industry, care facility, other. |
| Country first affiliation and name of the first affiliation | Report the country of the first affiliation listed and the name of the institute. |
| Conflict of interest statement reported | Was a conflict of interest statement reported? Answer: Yes/No/Unclear. If No, give the rationale. |
| No potential conflict of interest and funding issues | Were there no potential conflict of interests or funding issues that could have affected the outcomes of the survey? Answer: Yes/No/Unclear. If No, give the rationale. |
| Registration/publication of the protocol | Was the protocol registered or published a priori? Answer: Yes/No/Unclear. If Yes, we will also extract where and when the protocol was registered. |
| Institutional Review Board (IRB) approval | Report whether the survey was approved by an IRB. Answer: Yes/No/Unclear. |
| Institutional Review Board (IRB) exemption | Report whether the survey received an exemption from IRB. Answer: Yes/No/Unclear. |
| Limitations reported | Did the authors report the limitations of the survey? Answer: Yes/No/Unclear. |
| To do or contact authors | Report what actions should be undertaken to obtain additional information and whether it is necessary to contact the authors and if so report what information should be obtained. |

*We will assign ‘Unclear’ when too few details were reported in the manuscript or additional files to make a judgment of assigning ‘Yes’ or ‘No’.

**Table 2. Data collection forms at the eligibility level in surveys on HA issues***

| Target population | Report the type of target population of the survey, e.g., first or corresponding author or any other author. The target population further refers to characteristics such as assistant, associate, or full professor or head of a department etc. |
| --- | --- |
| Target field and context | Report the target field and context of the survey. For the field we refer to e.g., neurology or plastic surgery. For the context we refer to articles, journals, and publication dates on which the target population was surveyed. |

*We will assign ‘Unclear’ when too few details were reported in the manuscript or additional files to make a judgment of assigning ‘Yes’ or ‘No’.

**Table 3. Data collection forms at the sampling level in surveys on HA issues***

| Sampling technique | Report what sampling technique was used, e.g., consecutive, i.e., consecutive subjects were sampled, random, non-random, open link on a website, cluster (multistage), sampling weights, strata etc. |
| --- | --- |

*We will assign ‘Unclear’ when too few details were reported in the manuscript or additional files to make a judgment of assigning ‘Yes’ or ‘No’.

**Table 4. Data collection forms at the survey methods level in surveys on HA issues***

| Survey delivery (Subitem 1) | Report the methods of survey delivery, e.g., email, post, telephone etc. |
| --- | --- |
| Incentives (Subitem 2) | Report whether incentives were given to surveyees to complete the questionnaires. Answer: Yes/No/Unclear. If Yes, give the type of incentives. |
| Timeframe (Subitem 3) | Report whether the timeframe between the year of publication of a research publication and the date of the survey on this publication was reported. Answer: Yes/No/Unclear. If Yes, give the time frame. |
| Multiple (desired) submissions of surveys by the same surveyee (Subitem 4) | Report whether the manuscript reported that the surveyee completed more than 1 survey, e.g., when surveyees who have published multiple articles in the eligible time span were asked to submit a questionnaire for each published article. Answer: Yes/No/Unclear. If Yes, report on these multiple submissions. |
| Methods to prevent multiple (undesired) submissions of surveys by the same surveyee (Subitem 5) | Report whether methods were implemented for preventing the submitting of more than one questionnaire by the same surveyee when he/she was invited to submit only one. Answer: Yes/No/Unclear. If yes, give the methods. |

*We will assign ‘Unclear’ when too few details were reported in the manuscript or additional files to make a judgment of assigning ‘Yes’ or ‘No’.

**Table 5. Data collection forms at the surveyee level in surveys on HA issues***

| Characteristics of the responding surveyees | Report distribution characteristics of responding surveyees such as:  Sociodemographic characteristics: distributions of age (measure of central tendency (mean, p50), measure of dispersion [SD, IQR]), Sex, Career level such as PhD students, seniority etc. Country, Number of published papers, Additional characteristics. |
| --- | --- |
| Characteristics of the non-responding surveyees | Were the characteristics of the non-responding surveyees defined? Answer: Yes/No/Unclear. |

*We will assign ‘Unclear’ when too few details were reported in the manuscript or additional files to make a judgment of assigning ‘Yes’ or ‘No’.

**Table 6. Data collection forms at the response rate level in surveys on HA issues**

| Number of emails with questionnaires on HA issues sent (N1) | The total number of emails with questionnaires on HA issues sent. |
| --- | --- |
| Number of emails with questionnaires on HA issues not bounced (N2) | The total number of emails with questionnaires on HA issues sent that had surveyees with valid email addresses. |
| Number of questionnaires for which the surveyee was available (N3) | The total number of emails with questionnaires sent to assess HA issues with surveyees with valid email addresses and for which the surveyee was available. Unavailability can be the result of, e.g., automated responses such as ‘out of office’, ‘study leave’, ‘on strike’, ‘vacation leave’, ‘maternity leave’ etc. |
| Number of partly or completely answered questionnaires (N4) | The total number of questionnaires on HA issues received back in which the questions were answered (either partial or completely). |
| Number of completely answered questionnaires (N5) | The total number of questionnaires on HA issues received back in which all questions were answered. |
| Overall response rates in questionnaires on HA issues | N4 or N5/N1, N2, or N3 |

**Table 7. Data collection forms for review item 1***

| Review item 1 defined | Was review item 1 defined? Answer: Yes/No/Unclear. |
| --- | --- |
| Definition review item 1 | Report the definition of review item 1. |
| Reporting of survey question to assess review item 1 | Did the survey (or any additional file) report the survey question to assess review item 1? Answer: Yes/No/Unclear. |
| Survey question to assess review item 1 | Report the survey question to assess review item 1. |
| Type of answering scale | Yes/No/Unclear, Likert type answers etc. |
| Validation of the survey question to assess review item 1 | Was the question to assess review item 1 validated (tested) a priori e.g., through pilot testing or used in previous surveys? Answer: Yes/No/Unclear. |
| Number of questionnaires that answered the question on review item 1 (N6) | The total number of questionnaires received back in which the question on review item 1 was answered. |
| Reporting of the response rate on review item 1 | Was the initial sample size and the number of questionnaires that answered the question on review item 1 reported? Answer: Yes/No/Unclear. Initial sample size refers to any initial sample size, i.e., N1, N2, N3, N4, or N5 |
| Response rate on review item 1 | N6/N1, N2, N3, N4 or N5 |
| Magnitude of the response rate on review item 1 | Was the response rate on review item 1 higher than 50%? (Bethlehem 2017) Answer: Yes/No/Unclear. |
| Number of questionnaires in which the respondents reported review item 1 (N7) | The number of questionnaires in which the respondents reported review item 1, i.e., perceiving other co-author(s) as honorary author(s) on a publication. |
| Prevalence of review item 1 (**Primary outcome**) | N7/N6 |
| Sample size on the prevalence of review item 1 | Was the sample size adequate for the prevalence statistic of review item 1? Answer: Yes/No. |
| Approach to statistical analysis for review item 1 | Report the approach to statistical analysis (regression, group comparisons) for review item 1. |
| Complete reporting of outcome measures on review item 1 | Were the complete outcome measures given for review item 1, i.e., were the numerators and denominators reported? Answer: Yes/No. |
| Weighting of the survey results for review item 1 | Were the results of the survey for review item 1 weighted, i.e., corrected for selective nonresponse? Answer: Yes/No/Unclear. If Yes, describe the methods. |
| Additional issues | Report whether additional issues on review item 1 could have affected outcomes. Answer: Yes/No/Unclear. If Yes, explain. |

*We will assign ‘Unclear’ when too few details were reported in the manuscript or additional files to make a judgment of assigning ‘Yes’ or ‘No’.

**Table 8. Data collection forms for review item 2***

| Review item 2 defined | Was review item 2 defined? Answer: Yes/No/Unclear. |
| --- | --- |
| Definition review item 2 | Report the definition of review item 2 |
| Reporting of survey question to assess review item 2 | Did the survey (or any additional file) report the survey question to assess review item 2? Answer: Yes/No/Unclear. |
| Survey question to assess review item 2 | Report the survey question to assess review item 2. |
| Type of answering scale | Yes/No/Unclear, Likert type answers etc. |
| Validation of the survey question to assess review item 2 | Was the question to assess review item 2 validated (tested) a priori e.g., through pilot testing or used in previous surveys? Answer: Yes/No/Unclear. |
| Number of questionnaires that answered the question on review item 2 (N8) | The total number of questionnaires received back in which the question on review item 2 was answered. |
| Reporting of the response rate on review item 2 | Was the initial sample size and the number of questionnaires that answered the question on review item 2 reported? Answer: Yes/No/Unclear. Initial sample size refers to any initial sample size, i.e., N1, N2, N3, N4, or N5 |
| Response rate on review item 2 | N8/N1, N2, N3, N4 or N5 |
| Magnitude of the response rate on review item 2 | Was the response rate on review item 2 higher than 50%? (Bethlehem 2017) Answer: Yes/No/Unclear. |
| Number of questionnaires in which the respondents reported review item 2 (N9) | The number of questionnaires in which the respondents reported review item 2, i.e., perceiving other co-author(s) as honorary author(s) on a publication. |
| Prevalence of review item 2* (**Primary outcome**) | N9/N8 |
| Sample size on the prevalence of review item 2 | Was the sample size adequate for the prevalence statistic of review item 2? Answer: Yes/No. |
| Approach to statistical analysis for review item 2 | Report the approach to statistical analysis (regression, group comparisons) for review item 2 |
| Complete reporting of outcome measures on review item 2 | Were the complete outcome measures given for review item 2, i.e., were the numerators and denominators reported? Answer: Yes/No. |
| Weighting of the survey results for review item 2 | Were the results of the survey for review item 2 weighted, i.e., corrected for selective nonresponse? Answer: Yes/No/Unclear. If Yes, describe the methods. |
| Additional issues | Report whether additional issues on review item 2 could have affected outcomes. Answer: Yes/No/Unclear. If Yes, explain. |

*We will assign ‘Unclear’ when too few details were reported in the manuscript or additional files to make a judgment of assigning ‘Yes’ or ‘No’.

Note: Data extraction forms to extract review items 3-5 will be based on the same format as those reported in tables 7 and 8.

**Additional file 2B**

**Quality checklist for surveys on HA items**

The 14-item quality checklist presented below will be used for each of the review outcomes (review items 1-5) of this systematic review. This implies that 5 checklists will be prepared. Seven (Items **2, 5, 6, 7, 8, 12, and 13**) of the 14-item quality checklist were considered ‘critical’ (See ‘Guidance for rating the overall confidence in the results of the survey’). All checklist items will be collected in Excel spreadsheets.

**Table. Quality checklist for surveys on review item (#)***

| **#** | **Item** | **Question** |
| --- | --- | --- |
| 1 | No conflict of interest and funding issues regarding review item (#) | Were there no potential conflict of interests or funding issues that could have affected the outcome of review item (#)? Answer: Yes/No/Unclear. If No, give the rationale. |
| 2 | Selective (non) reporting regarding review item (#) | Was there no risk of selective (non-) reporting bias regarding review item (#)? For example: (1) non registering or publication of the review protocol (2) incomplete reporting on the outcomes of review item (#) or changes in definitions of this review item or changes in analyses of this review item that were not congruent with those planned. Answer: Yes/No/Unclear. If No, give the rationale. |
| 3 | Target population, field, and context for review item (#) defined | Were the target population, field, and context for the survey on review item (#) defined? Answer: Yes/No/Unclear. |
| 4 | Sampling for review item (#) | Did each individual in the target population have an equal chance for being selected for the survey on review item (#)? Answer: Yes/No/Unclear. |
| 5 | Survey methods for review item (#)** | Were there no survey methods that could have introduced bias, i.e., systematic error in the outcomes of the survey on review item (#)? Answer: Yes/No/Unclear. If No, give rationale how bias was introduced. |
| 6 | Responding surveyees for review item (#) defined | Were the characteristics of the responding surveyees on review item (#) defined? Answer: Yes/No/Unclear. |
| 7 | Responding surveyees for review item (#) representative for the target population | Were the characteristics of the responding surveyees on review item (#) representative for the target population ? Answer: Yes/No/Unclear. |
| 8 | Review item (#) defined | Was review item (#) defined? Answer: Yes/No/Unclear. |
| 9 | Reporting of survey question to assess review item (#) | Did the survey (or any additional file) report the survey question to assess review item (#)? Answer: Yes/No/Unclear. |
| 10 | Validation of the survey question to assess review item (#) | Was the question to assess review item (#) validated (tested) a priori e.g., through pilot testing or used in previous surveys? Answer: Yes/No/Unclear. |
| 11 | Reporting of the response rate on item (#)*** | Was the initial sample size and the number of questionnaires that answered the question on review item (#) reported? Answer: Yes/No/Unclear. Initial sample size refers to any initial sample size, i.e., N1, N2, N3, N4, or N5 |
| 12 | Response rate on review item (#)** | Did the magnitude of the response rate on review item (#) or the way the response rate (in the case of a low response rate) was managed provide certainty in the validity of the results on this review item?  Answer: Yes/No/Unclear. |
| 13 | Sample size on the prevalence of review item (#)** | Was the sample size adequate for the prevalence statistic of review item (#)? Answer: Yes/No/Unclear. |
| 14 | Complete reporting of outcome measures on review item (#) | Were the complete outcome measures given for review item (#), i.e., were the numerators and denominators reported? Answer: Yes/No/Unclear. |

*We will assign ‘Unclear’ when too few details were reported in the manuscript or additional files to make a judgment of assigning ‘Yes’ or ‘No’.

**Guidance for addressing this question is reported under here in the section ‘Guidance for completing the checklist for surveys on HA issues’.

***Definitions of all samples are given in Table 3 of the main manuscript.

**Guidance for completing the quality checklist for surveys on HA issues**

Answering most items is straightforward. Items that need additional guidance are presented under here.

**Item 3. Target population, field, and context for review item defined**

The target population refers to the characteristics of the surveyee, i.e., first or corresponding author or any other author. The target population further refers to characteristics such as assistant, associate, or full professor or head of a department etc. The target field refers to e.g., neurology or plastic surgery. For the context we refer to articles, journals, and publication dates on which the target population was surveyed.

**Item 5. Survey methods**

For this item we will consider 5 subitems: 1) survey delivery; 2) incentives; 3) timeframe; 4) multiple (desired) submissions by the same surveyee; 5) methods to prevent multiple (undesired) submissions of surveys by the same surveyee. We will first address the signaling questions for each subitems. Based on the answers to these signaling questions we will address the checklist question. ‘No’ will be assigned when bias could be the result of methodological issues in one or more of these subitems, which could lead to systematic error in the outcomes of the survey. When ‘No’ is scored we will also give the rationale.

**Table. Signaling questions for Item 5. Survey methods***

| **Subitem** | **Signaling questions** |
| --- | --- |
| Survey delivery (Subitem 1) | Were the methods of survey delivery reported, e.g., email, post, telephone etc. Answer: Yes/No/Unclear. If Yes, give the type of survey delivery. |
| Incentives (Subitem 2) | Report whether incentives were given to surveyees to complete the questionnaires. Answer: Yes/No/Unclear. If Yes, give the type of incentives. |
| Timeframe (Subitem 3) | Report whether the timeframe between the year of publication of a research publication and the date of the survey on this publication was reported. Answer: Yes/No/Unclear. If Yes, give the time frame. |
| Multiple (desired) submissions of surveys by the same surveyee (Subitem 4) | Report whether the manuscript reported that the surveyee completed more than 1 survey, e.g., when surveyees who have published multiple articles in the eligible time span were asked to submit a questionnaire for each published article. Answer: Yes/No/Unclear. If Yes, report on these multiple submissions. |
| Methods to prevent multiple (undesired) submissions of surveys by the same surveyee (Subitem 5) | Report whether methods were implemented for preventing the submitting of more than one questionnaire by the same surveyee when he/she was invited to submit only one. Answer: Yes/No/Unclear. If yes, give the methods. |

*We will assign ‘Unclear’ when too few details were reported in the manuscript or additional files to make a judgment of assigning ‘Yes’ or ‘No’.

**Item 12. Response rate**

The impact of nonresponse on the results of a survey will be little when response rates are high (Bethlehem 2017), but low response rates may diminish the validity of a survey’s results. However, ‘Yes’ can still be answered to the question in item 12 when response rates are modest, i.e., when authors can show that non-response was not related to the outcome measured and that the characteristics of responders and non-responders are comparable. Whether and how adjustment weighting was implemented will be considered when answering the question of item 12. Adjustment weighting refers to correcting for selective nonresponse. For example, assigning higher weights to underrepresented respondents. In this context we will also consider the issue of same surveyees submitting more than one questionnaire, e.g., when surveyees who have published multiple articles in the eligible time span for the survey were asked to submit a questionnaire for each published article.

**Item 13. Sample size**

We will calculate the required sample size with EpiTools epidemiological calculators based on the identified prevalence and the total sample size (Sergeant 2018). The estimated prevalence will be calculated with a 0.95 confidence level (desired precision of estimate 0.05).

**Guidance for rating the overall confidence in the results of a survey**

Our rating of the overall confidence in the results of a survey reflects how non-implementation of one or more of these 14 safeguard items might possibly have impacted bias of the results of the survey. Seven (Items 2, 5, 6, 7, 8, 12, and 13) of the 14-item checklist were considered ‘critical’ for this rating. We adopted the rating scheme reported for the AMSTAR 2 critical appraisal tool (Shea 2017) to assign ratings of the overall confidence in the results of a survey. Table 1. presents this rating scheme and is an exact copy of the AMSTAR 2 instrument.

**Table 1.** **Rating the overall confidence in the results of a survey***

| **Rating** | **Description** |
| --- | --- |
| High | No or one non-critical weakness was scored in the 14-item quality checklist |
| Moderate | More than one non-critical weakness* was scored in the 14-item quality checklist |
| Low | One critical flaw with or without non-critical weaknesses was scored in the 14-item quality checklist |
| Critically low | More than one critical flaw with or without non-critical weaknesses was scored in the 14-item quality checklist |

*Multiple non-critical weaknesses may diminish confidence in a survey and it may be appropriate to move the overall appraisal down from moderate to low confidence

**Tabular presentation for rating the overall confidence in the results of review item (#)**

**Table 2. Tabular presentation of the scores of the 14 item quality checklist for review item (#)* ****

| **Reference** | **Q1** | **Q2** | **Q3** | **Q4** | **Q5** | **Q6…etc. Q14** | **Overall confidence in the results** |
| --- | --- | --- | --- | --- | --- | --- | --- |
|  |  |  |  |  |  |  |  |

* All critical appraisal scores Yes/No/Unclear will be given for each outcome

** Overall confidence ratings are: High, Moderate, Low, and Critically low

**Guidance for grading the certainty or quality of evidence for a review item**

We will use the GRADE approach (Schünemann 2021) for grading the certainty or quality of evidence for each of the 5 outcomes (review items 1-5) of our planned systematic review on survey research. The GRADE approach assigns four levels of certainty: ‘High’, ‘Moderate’, ‘Low’, and ‘Very low certainty’ that a point estimate for a specific outcome is correct (Schünemann 2021). The rationale for assigning these ratings for each outcome will be given.

GRADE ratings for outcomes of interventional studies start with assigning high quality to randomized controlled trials and low quality to observational studies. For surveys we will assign high quality when surveyees had an equal chance of being selected for the survey and low quality when they did not. We will start with this initial quality rating and will then according to the GRADE approach assess 5 factors that can lower the quality rating. These 5 factors are presented as domains 1-5 and are explained.

Domain 1. Risk of bias

Domain 2. Inconsistency

Domain 3. Indirectness

Domain 4. Imprecision

Domain 5. Publication bias

**Domain 1. Bias in the included surveys.**

Bias in the included surveys will be based on our 14-item quality checklist reported in Additional file 2B. For each outcome we will assign one of the following overall confidence ratings: High, Moderate, Low, and Critically low. (See quality checklist for surveys on HA items in Additional file 2B). The rationale for assigning each type of rating will be given for each outcome.

**Domain 2. Heterogeneity or inconsistency of results.**

We will assess the presence and the extent of heterogeneity. In the forest plots we will assess the overlap of the confidence intervals for the results of the individual surveys. We will calculate Tau^2^ (Estimate of between study variance) and Chi^2^ tests to measure statistical heterogeneity (Deeks 2021). We will calculate I^2^ to quantify inconsistency and will use the following rough interpretation of pertinent thresholds for I^2^ (Deeks 2021).

0% to 40%**:** might not be important

30% to 60%: may represent moderate heterogeneity

50% to 90%: may represent substantial heterogeneity

75% to 100%: considerable heterogeneity

**Domain 3. Indirectness of evidence**

Indirectness of evidence will be assigned when for example not all, but only a subgroup of corresponding authors (for example only the heads of departments) of a target population were surveyed. Outcomes for such a subgroup will not be representative for all corresponding authors of that target population.

**Domain 4. Imprecision of results**

Surveys with few surveyees or with few events are imprecise and will have wide confidence intervals (Schünemann 2021). We will assess imprecision for each outcome.

**Domain 5. Publication bias**

For this domain we will assess whether publication bias is likely. Methods to detect publication bias will include: asymmetrical funnel plots, small studies, selective availability of data from published or non-published surveys (Schünemann 2021).

**Additional file 2C**

**Summary of findings tables**

Tables with the characteristics of included surveys on HA issues and the pertinent response rates in these surveys will be based on the collected items in tables 1-8 of the data collection forms (Additional file 2A). Further, summary of findings tables will be prepared that represent the characteristics of the question and the outcomes for each of the 5 review items. One exemplary table for review item 1 is presented under here. For each of the 5 review items similar tables will be presented in the final manuscript.

**Table 1. Characteristics of question on review item 1**

| **Reference** | **Definition** | **Survey question** | **Type of answering scale** | **Validation of the survey question** |
| --- | --- | --- | --- | --- |
|  |  |  |  |  |

**Table 2. Tabular presentation of the scores of the 14-item quality checklist for review item 1* ****

| **Reference** | **Q1** | **Q2** | **Q3** | **Q4** | **Q5** | **Q6…etc. Q14** | **Overall confidence in the results** |
| --- | --- | --- | --- | --- | --- | --- | --- |
|  |  |  |  |  |  |  |  |

* All critical appraisal scores Yes/No/Unclear will be given for each outcome

** Overall confidence ratings are: High, Moderate, Low, and Critically low

**Table 3. Outcomes on review item 1 and confidence in the cumulative evidence**

| **Reference** | **Number of questionnaires that answered the question on review item 1 (N6)** | **Response rate on review item 1 (N6/N1, N2, N3, N4, or N5)** | **Number of questionnaires in which the respondents reported review item 1 (N7)** | **Prevalence of review item 1 (Primary outcome)**  **(N7/N6)** |
| --- | --- | --- | --- | --- |
| **Confidence in the cumulative evidence (GRADE approach)*** |  |  |  |  |

*The GRADE approach assigns four levels of certainty: ‘High’, ‘Moderate’, ‘Low’, and ‘Very low certainty’ (Schünemann 2021). The rationale for assigning these ratings will be given.

**References for additional file 2**

**Bethlehem 2017**

Bethlehem J.  Chapter 12: A checklist for polls. In ‘Understanding Public Opinion Polls’. Boca Raton, Florida (USA): Chapman and Hall/CRC; 2017. ISBN: 978-1498769747.

**Deeks 2021**

Deeks JJ, Higgins JPT, Altman DG (editors). Chapter 10: Analysing data and undertaking meta-analyses. In: Higgins JPT, Thomas J, Chandler J, Cumpston M, Li T, Page MJ, Welch VA (editors). Cochrane Handbook for Systematic Reviews of Interventions version 6.2 (updated February 2021). Cochrane, 2021. Available from [www.training.cochrane.org/handbook](http://www.training.cochrane.org/handbook).

**Schünemann 2021**

Schünemann HJ, Higgins JPT, Vist GE, Glasziou P, Akl EA, Skoetz N, Guyatt GH. Chapter 14: Completing ‘Summary of findings’ tables and grading the certainty of the evidence. In: Higgins JPT, Thomas J, Chandler J, Cumpston M, Li T, Page MJ, Welch VA (editors). Cochrane Handbook for Systematic Reviews of Interventions version 6.2 (updated February 2021). Cochrane, 2021. Available from [www.training.cochrane.org/handbook](http://www.training.cochrane.org/handbook).

**Sergeant 2018**

Sergeant ESG. Sergeant, ESG, 2018. Epitools Epidemiological Calculators. Ausvet. [online] Available from: [http://epitools.ausvet.com.au](http://epitools.ausvet.com.au/). (accessed April 10^th^ 2021).

**Shea 2017**

[Shea BJ](https://www.ncbi.nlm.nih.gov/pubmed/?term=Shea%20BJ%5BAuthor%5D&cauthor=true&cauthor_uid=28935701), [Reeves BC](https://www.ncbi.nlm.nih.gov/pubmed/?term=Reeves%20BC%5BAuthor%5D&cauthor=true&cauthor_uid=28935701), [Wells G](https://www.ncbi.nlm.nih.gov/pubmed/?term=Wells%20G%5BAuthor%5D&cauthor=true&cauthor_uid=28935701), [Thuku M](https://www.ncbi.nlm.nih.gov/pubmed/?term=Thuku%20M%5BAuthor%5D&cauthor=true&cauthor_uid=28935701), [Hamel C](https://www.ncbi.nlm.nih.gov/pubmed/?term=Hamel%20C%5BAuthor%5D&cauthor=true&cauthor_uid=28935701), [Moran J](https://www.ncbi.nlm.nih.gov/pubmed/?term=Moran%20J%5BAuthor%5D&cauthor=true&cauthor_uid=28935701), [Moher D](https://www.ncbi.nlm.nih.gov/pubmed/?term=Moher%20D%5BAuthor%5D&cauthor=true&cauthor_uid=28935701), [Tugwell P](https://www.ncbi.nlm.nih.gov/pubmed/?term=Tugwell%20P%5BAuthor%5D&cauthor=true&cauthor_uid=28935701), [Welch V](https://www.ncbi.nlm.nih.gov/pubmed/?term=Welch%20V%5BAuthor%5D&cauthor=true&cauthor_uid=28935701), [Kristjansson E](https://www.ncbi.nlm.nih.gov/pubmed/?term=Kristjansson%20E%5BAuthor%5D&cauthor=true&cauthor_uid=28935701), [Henry DA](https://www.ncbi.nlm.nih.gov/pubmed/?term=Henry%20DA%5BAuthor%5D&cauthor=true&cauthor_uid=28935701). AMSTAR 2: a critical appraisal tool for systematic reviews that include randomised or non randomised studies of healthcare interventions, or both. [BMJ.](https://www.ncbi.nlm.nih.gov/pubmed/?term=AMSTAR+2%3A+a+critical+appraisal+tool+for+systematic+reviews+that+include+randomised+or+non-randomised+studies+of+healthcare+interventions%2C+or+both) 2017 Sep 21;358:j4008. doi: 10.1136/bmj.j4008.
